# Implantation and Analysis of a Biphasic Bioinspired Osteochondral Chitosan Construct in a Large Animal Model

**DOI:** 10.1021/acsbiomaterials.5c01821

**Published:** 2026-03-13

**Authors:** Laura Macri Pellizzeri, Jane S. McLaren, Reda Felfel, Katherine Pitrolino, Robert Kerslake, Colin Scotchford, David M. Grant, Brigitte S. Scammel, Virginie Sottile

**Affiliations:** † School of Medicine, 6123 University of Nottingham, Nottingham NG7 2UH, U.K.; ‡ Advanced Materials Research Group, Faculty of Engineering, University of Nottingham, Nottingham NG7 2RD, U.K.; § Department of Mechanical and Aerospace Engineering, University of Strathclyde, Glasgow G1 1XJ, U.K.; ∥ Department of Physics, Faculty of Science, Mansoura University, Mansoura 35516, Egypt; ⊥ Research and Innovation Office, Loughborough LE11 3TU, U.K.; # Department of Molecular Medicine, 19001 University of Pavia, Pavia 27100, Italy; ∇ UOC Bioscaffolds and Transplantation, Fondazione IRCCS Policlinico San Matteo, Pavia 27100, Italy

**Keywords:** osteochondral repair, scaffold, biomaterial, regenerative medicine, large animal model

## Abstract

Osteochondral defects are severe degenerative lesions of the joint that involve the articular cartilage, the cartilage–bone interface, and the underlying subchondral bone. Surgical approaches that have been developed (joint debridement, microfracture, autologous chondrocytes and autologous/allograft cartilage implantation, and whole joint replacement) remain, however, more palliative than curative. Therefore, novel strategies are required, including promising options offered by osteochondral tissue engineering approaches. In this study, a novel chitosan-based scaffold with biomimetic and biodegradable properties was implanted for 16 weeks in a sheep osteochondral defect model in order to evaluate its physical stability, biocompatibility, and regenerative potential. Alongside the chitosan construct, a commercial scaffold made of collagen and ß-TCP was implanted as a positive control, while defects left empty were used as negative control. Postoperative recovery and tissue response were evaluated at different levels including activity monitoring, gross tissue evaluation at sacrifice, imaging, and histological analysis. Despite a transient reaction observed in the first 4 weeks after implantation, the chitosan-based scaffold was well-tolerated, with no clear signs of local or systemic reaction in the long term. The chitosan scaffold was able to support the growth of mature bone tissue with a lamellar conformation at similar levels to the predicate control. It also promoted the formation of a new cartilage layer, although not at a fully mature stage. These proof-of-concept results suggest that the novel chitosan scaffold tested constitutes a possible option for osteochondral regeneration, which should be further analyzed in complementary models to provide information on its long-term performance.

## Graphical abstract

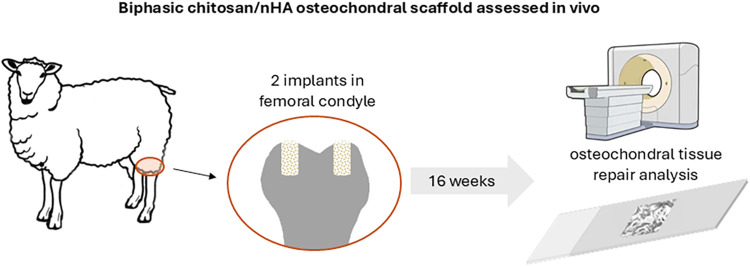

## Introduction

Osteochondral defects (OCDs) are severe degenerative lesions of the joint that involve the layer of articular cartilage, the cartilage–bone interface, and the underlying subchondral bone. OCDs are associated with significant pain and impaired locomotion ability. The incidence of OCDs is increasing due to the aging of the population, which is more likely susceptible to develop osteoarthritis, one of the main degenerative conditions associated with OCDs.^1^ A number of surgical approaches have been developed over the years, including joint debridement, microfracture, autologous chondrocytes, and autologous/allograft cartilage implantation to whole joint replacement. Although they have been associated with some pain relief and improved life quality, these interventions do not yet lead to the complete healing of the lesions and remain mainly palliative.^2^ The main challenge in the regeneration of OCDs resides in the complexity of the osteochondral tissue composed of bone and cartilage, which require specific soluble and mechanical stimuli.^2^ While resident mesenchymal precursor cells are recruited and lead the in vivo regeneration of the subchondral bone, the regeneration of the above cartilage layer is challenging due to its avascular nature and complex geometry.^3^ In this sense, osteochondral tissue engineering using biomimetic materials has arisen as a promising strategy to provide a stable and life-long treatment for OCDs. Biomimetic scaffolds can be made of bioactive materials, possibly enriched with precursor cells and/or growth factors to stimulate and guide the endogenous reparative processes of the body.^4^ Numerous materials have been developed over recent years to produce biphasic scaffolds for osteochondral tissue engineering, including poly­(lactide-*co*-glycolide), silk fibroin, or collagen, combined with calcium-phosphate formulations.^5^ Another biomaterial of interest is chitosan, a long-chain biopolymer with appealing features such as biocompatibility, tuneability for a more tailored approach, biodegradability, and chemical similarity to the glycosaminoglycans naturally occurring in articular cartilage.^6^ Over recent years, the structural and chemical properties of chitosan shown to favor cell adhesion and interactions have been noted as relevant for tissue engineering, and its versatility in terms of manufacturing, functionalization, and drug-loading has led to different types of constructs being proposed for orthopedic applications.^7^ Importantly, chitosan use is already approved in the clinic for its intrinsic hemostatic properties, for instance as a commercial chitosan-based bandage (HemCon Patch).^8^

The present study investigated the in vivo biocompatibility and regenerative potential of a new biomimetic chitosan-based scaffold functionalized with nanohydroxyapatite (nHA) rods, implanted in a critical size osteochondral defect in sheep. This continuous biphasic construct is a scaffold that mimics both the cartilage and subchondral bone regions in terms of geometry, porosity, and nHA incorporation.^9^ The physical continuity between the two phases confers stability as established in previous mechanical tests^9^ and avoids delamination, which occurs in osteochondral multilayered implants due to the shear stress and mechanical loads experienced in the joint, and can compromise the clinical success.^10^ Functionalization with HA improves mechanical properties and promotes osteogenic regeneration due to the osteoinductive and osteoconductive properties of HA,^11^ as examined in previous in vitro studies with human mesenchymal progenitors.^9^ Implantation in a sheep model was performed to determine the scaffold’s ability to remain in position without delamination and to support defect filling with new bone-like and cartilage-like tissue over the period of study.

## Experimental Section

### Scaffold Preparation

Bilayered chitosan scaffolds were manufactured using a combination of porogen leach-out (polycaprolactone (PCL) microspheres were used as porogen) and freeze-drying approaches. A chitosan (molecular weight of 471 kDa and degree of deacetylation of 84% ± 2%) solution (4%) was prepared in distilled water with 2.5 v/v% glacial acetic acid and kept overnight to remove any trapped air bubbles. The scaffolds were chemically cross-linked using 2.5 w/w% of a natural cross-linker genipin. The first layer contained PCL microspheres at a particle size range of 300–425 μm, while PCL microspheres at a particle size range of 180–300 μm were used in the second layer. The bone-like side was made of a chitosan–hydroxyapatite (nHA) nanocomposite, a suspension of nHA rods were incorporated at 70 wt % in the chitosan solution, whereas the chondrogenic side was made of chitosan alone. Individual scaffolds were cut from the mold using a 8.5 mm corkborer, and the height was adjusted to 8 mm. The scaffolds were kept individually in plastic vials and packed in Tyvek sterilization pouches (Westfield Medical Ltd., UK) and irradiated according to BS EN ISO 11137-2 standard with a dose of 25 ± 2.5 kGy using a ^60^Co γ-ray source (JRI Orthopedics). Scaffolds remained in sterilization pouches until use.

### In Vivo Bone Defect Model

All procedures were carried out in compliance with the UK Home Office regulations after approval from the University of Nottingham Animal Welfare and Ethical Review Body. Nine skeletally mature English mule sheep were randomly allocated to three groups:

- Chitosan construct group, implanted with the monophasic bilayered chitosan implant manufactured as above;
- Predicate construct group, implanted with a commercial biphasic implant made of bovine collagen type I in the chondral phase and of 80% ß-TCP, 20% PLA in the subchondral phase (BioMatrix CRD, Arthrex, USA);
- Empty group, with the defect left empty used as a negative control.

Animals were screened to ensure good physical condition and mobility and were acclimatized to the new environment for a minimum of 7 days prior to surgery. Anesthesia was induced with Ketaset (ketamine, 2 mg/kg, Fort Dodge Animal Health Ltd., Southampton, UK) and 2.5 mg Hypnovel (Midazolam, Roche Products Ltd., Welwyn Garden City, UK), and then maintained with 2% isoflurane (Abbott Laboratories Ltd., Maidenhead, UK) in 100% oxygen. Pre- and postoperative analgesia was given via sustained release Durogesic patches (fentanyl, 2 μg/kg/h, 12 h before surgery and for at least 6 days after surgery, Janssen-Cilag, Saunderton, UK). If additional analgesia was required, buprenorphine was injected intramuscularly every 12 h. Penicillin and streptomycin were given as a prophylactic antibiotic (Pen & Strep Suspension for injection, Norbrook, Newry, Northern Ireland; 1 mL/25 kg intramuscularly) preoperatively and 24 h postoperatively. During surgery, animals were placed in lateral recumbency to allow access and the area was then clipped and prepared using chlorhexidine gluconate solution BP 20% (Hibiscrub Veterinary, Schering-Plough, UK). Marcaine (Dechra, Shrewsbury, UK) was given as a local anesthetic in the area of the incision. Once in theater, the area was thoroughly washed with Virusan, an ethanol-based sanitizer containing chlorhexidine gluconate (Cairn Technology, Sheffield, UK). A stomach tube was placed for the duration of the surgery and Hartmann’s solution (Vetivex 11, Dechra, Shrewsbury, UK) was given intravenously.

Each animal received treatment in two defects made within the left hind leg sealed joint capsule (in the lateral and medial condyles). This allowed us to avoid confounding the data with two different products within the same joint capsule and maintain one untreated hind leg for support. To access both the lateral and medial condyles, a skin incision was made over the patella tendon before initially creating the defect in the medial condyle (Figure S1A). A medial parapatellar incision was made with patella retracted but not luxated until the condyle was exposed. A cylindrical 8 mm diameter and 8 mm depth defect was created on the weight-bearing surface. Drilling began using a 2 mm drill bit and progressed in 2 mm increments using a power drill until finally the 8 mm defect was created using a T handle hand drill to provide additional control, followed by a reamer to ensure a flat defect base. All drill bits and the reamer had 8 mm stops to ensure a standardized defect size. During drilling and reaming, the drills were cooled with a sterile saline solution. A depth gauge was used to confirm the defect depth before rinsing with saline and packing with a saline-soaked swab while the lateral defect was created. A lateral parapatellar incision was created with patella retracted but not luxated, and an identical 8 mm defect was made with the same drill increments. The predicate preparation was performed following the manufacturer’s instructions before implantation. This involved placing the predicate construct in the delivery device and wetting it on a tray containing sterile saline for 5 min before implantation (Figure S1B). The chitosan product was wetted with sterile saline and degassed^12^ before loading into the delivery device (Figure S1C). For the empty group, the defects were left empty. At the end of the intervention, the joint capsule was sutured at both the medial and lateral incisions using cruciate stitches (Vicryl 2, Ethicon, Kirkton, UK). The subcutaneous tissue and skin were closed using resorbable subcuticular sutures (Vicryl 1, Ethicon, Kirkton, UK). Marcaine was reapplied to the defect site to provide additional local analgesia. A permeable-to-moisture vapor spray dressing (Opsite, Smith & Nephew Healthcare, Hull, UK) was then applied to protect the wound.

Clinical and posture and locomotion scoring was carried out for a minimum of 6 days postoperation and activity monitors (FitBark Inc., USA) were fixed on collars to monitor physical activity for the entire 16-week study. At 16 weeks, the sheep were sacrificed using an overdose of barbiturate (pentobarbital solution 20%, 0.7 mg/kg, Dolethal, Vétoquinol UK Ltd., Buckingham, UK). Entire stifle joints were harvested and placed in 10% neutral buffered formalin (NBF, Sigma-Aldrich, Gillingham, UK) for a minimum of 7 days before further processing. Joints from the unoperated legs were used as controls.

### Macroscopic Observation and Scoring

All fixed condyles were observed for macroscopic scoring of cartilage repair performed according to a defined set of criteria (Table 1) adapted from the ICRS and Goebel systems.^13,14^ The maximum and minimum scores assignable were 16 and 0, respectively, with higher scores indicating better regeneration. Scoring was performed blind by three operators observing either the samples *ex vivo* or sample pictures acquired by using a reflex camera (Nikon D3600).

**Table 1:** Scoring System Used for the Macroscopic Evaluation of Cartilage Repair^a^

category		point
degree of defect repair (surface): *From ICRS*	100%	4
	>75% repair of defect surface	3
	75–50% repair of defect surface	2
	50–25% repair of defect surface	1
	25–0% repair of defect surface	0
integration to host tissue: *From ICRS*	complete integration with surrounding cartilage	4
	demarcating border (<1 mm)	3
	3/4 of graft integrated, 1/4 with a notable border (>1 mm)	2
	1/2 of graft integrated, 1/2 with a notable border (>1 mm)	1
	from no contact to 1/4 of graft integrated with surrounding cartilage	0
macroscopic appearance: *From ICRS*	smooth surface	4
	fibrillated surface	3
	small, scattered fissures or cracks	2
	several, small or few but large fissures	1
	not regeneration of grafted area	0
color: *From Goebel*	hyaline or white	4
	predominantly white (>50%)	3
	predominantly translucent (>50%)	2
	translucent	1
	no repaired tissue	0
maximum score		16

a Adapted from ICRS and Goebel systems.

### Magnetic Resonance Imaging (MRI)

MRI was performed on fixed femoral samples washed in PBS for 7 days to remove any residual fixative. PBS was made in-house dissolving NaCl (137 mM), KCl (2.7 mM), Na_2_HPO_4_·2H_2_O (10 mM), and KH_2_PO_4_ (1.8 mM) in distilled water and pH adjusted to 7.4. PBS was refreshed every day for 7 days. Before the scan, the bones were wrapped in parafilm to prevent them from drying out. A 0.5 mL centrifuge tube was positioned on the medial side for reference. Samples were scanned in a 7 T Bruker Avance III system using Paravision 6 (Bruker Biospin, Ettlingen, Germany). Images were collected using the Bruker fast spin–echo protocol known as RAREst with the following parameters: effective TE = 8.22 ms, echo spacing = 8.215 ms, repetition time = 30 s, echo train length = 1, field-of-view = 102.4 × 72 mm, matrix size = 512 × 360, and slice thickness = 0.21 mm. A total of 256 slices were collected using 8 signal averages in a single scan lasting 1.3 h. After imaging, the samples were returned to the fixative solution until further processing. Images were blindly assessed and scored on the basis of predefined criteria (Table 2) by two independent operators.

**Table 2:** Scoring System for the Evaluation of Tissue Response in MRI and mCT Scans^a^

	score				
parameter	0	1	2	3	4
depth of filling surface	significantly lower, not continuous with the endogenous sides	significantly lower but anchored to the endogenous tissue	slightly lower	flush	
new bone	no new bone	new bone only at bottom/side/subchondral, possibly lower density and poorer structure than endogenous tissue	new bone in 2 of the 3 regions (bottom, sides, subchondral)	new bone filling almost the entire defect, possible open collar at the subchondral region	new bone filling almost the entire defect, highly mineralized closed collar at subchondral region
soft/fibrous tissue	spread throughout the defect	soft tissue pocket with a remarkable size	soft tissue pocket with a small size	no soft tissue	
cartilage	no cartilage-like tissue	cartilage-like only on the edges toward the endogenous tissue, gap in the midline	cartilage-like layer, mainly uneven, thickness different than the endogenous tissue	cartilage-like layer, mainly even (possible small invagination), thickness slightly lower than the endogenous tissue	cartilage-like layer, even, reaching from side to side, no invaginations and thickness similar to the endogenous tissue
original edges of the defect (mCT images)	clearly visible all around the defect (suggesting no new tissue ingrowth)	visible for most of the defect (tissue ingrowth only in limited areas)	visible only at the entrance of the defect	not visible	

a Maximum score = 17.

### Microcomputed Tomography Analysis (Micro-CT)

Microcomputed tomography (micro-CT) analysis was performed to quantify bone formation within the defect. Scans were performed with a high-resolution micro X-ray computed tomography system (micro-CT, Skyscan 1174, Bruker) using the following conditions: 50 kV voltage, 800 μA current, voxel resolution of 32 μm,^15,16^ and application of a 0.50 μm aluminum filter. A threshold of 255/50 was selected to segment bone from the surrounding tissue. Transmission images were reconstructed using Skyscan supplied software (NRecon) with the resulting two-dimensional image representing a single 32 μm slice (1/256).

### Tissue Processing, Histological Staining, and Assessment

Before the decalcification began, the defects were halved on the longitudinal plane. Samples were decalcified in 10% ethylenediaminetetraacetic acid solution (EDTA, Sigma-Aldrich) at pH 7.4 under gentle agitation for 12 weeks. The solution was refreshed once a week, and the decalcification progress was monitored through X-ray imaging every 4 weeks using a portable X-ray unit (HiRay Plus, Eickemeyer, UK). Fully decalcified samples were wax-embedded and sectioned. Each half defect was further cut in 5 regions (L1–L5) from the center toward the edge (Figure S2). Each region was then sectioned into 5 μm slices. For histological staining, sections were dewaxed, rehydrated, and stained with hematoxylin & eosin, 1% w/v Alcian blue at pH 2.5, or Masson’s trichrome staining (Merk, DE). Stained sections were imaged on a Nikon Eclipse LV100ND microscope (Nikon Instruments, JP) coupled with a Nikon digital sight DS-Ri1 camera and a NIS Elements software (version 4.1).

Histological scoring was performed on Alcian blue stained sections from the L1 and L2 regions using a set of defined parameters (Table 3). For the scoring of each defect, 3 different areas were selected: one in the center of the defect (“midline”) and two areas on either side of the defect midline (defined as “right side” and “left side”), which included the border region of the defect with the endogenous tissue (Figure S2). The maximum score assignable was 36 arbitrary units (a.u.) for the right/left sides (9 parameters included), and 34 au for the midline area (8 parameters included), with a higher score indicating better regeneration.

**Table 3:** Histological Scoring System Used to Evaluate the New Cartilage-like Tissue in Alcian Blue Stained Sections^a^

parameter
(1) Is there a layer of cartilage-like tissue covering the defect?
– yes: 4 pts
– no: 0 pts
(2) Which is the thickness?
– the same as endogenous tissue (100% of endogenous tissue): 4 pts
– thinner/thicker: 2 pts
(3) Is it at the same level of the endogenous tissue?
– yes (even or <10% lower than adjacent tissue): 4 pts
– no:
– slightly lower (10–50% of the thickness lower): 3 pts
– clearly lower (50–100% of the thickness lower): 1 pts
– significantly lower (>100% of the thickness lower): 0 pt
(4) How does the cartilage look like?
– flat, intact and smooth: 4 pts
– rough and irregular: 2 pts
– it presents horizontal laminations and fissures: 1 pt
– it is severely disrupted displaying fibrillations: 0 pt
(5) Nature of cartilage:
– mostly hyaline: 4 pts
– mixed: 3 pts
– mainly fibrocartilaginous: 2 ps
– all fibrocartilaginous: 1 pt
– not cartilaginous: 0 pt
(6) Metachromasia I—presence of staining:
– yes: 4 pts
– no: 0 pt
(7) Metachromasia II—intensity of the staining:
– strong: 4 pts
– pale: 2 pts
(8) Metachromasia III—quality of the staining:
– homogeneous: 4 pts
– heterogeneous: 2 pts
(9) Continuity with endogenous cartilage:
*(right and left sides only)*
– bonded: 4 pts
– not bonded: 0 pt

a For the scoring of the right and left sides, the parameters 1–9 were used, while for the midline defect area the scoring was performed according to parameters 1–8.

### ELISA Semiquantitation of C-Reactive Protein Levels

C-reactive protein (CRP) levels were assayed in serum samples collected at day 0 (before surgery), day 7, and before sacrifice at the end point (16 weeks). Blood samples were collected from the jugular vein and allowed to coagulate overnight at 4 °C. Samples were centrifuged at 2000*g* for 10 min at 4 °C, and serum was aliquoted and frozen at −20 °C. For CRP semiquantitation, a CRP ELISA test kit (Abclonal) was used following manufacturer’s instructions, and the optical density was measured at 450 nm in a microplate reader (Tecan, CH).

### Statistical Analysis

After confirming that data was normally distributed, the micro-CT data were analyzed using a one-way ANOVA, while for the histological scoring, a two-way ANOVA test was applied. A posthoc Tukey test was applied to both the micro-CT data and the histological scoring data. All statistical analyses were carried out using GraphPad PRISM version 9.0.2, and all data are shown as mean values ± the standard error of the mean (SEM).

## Results

### Postoperative Monitoring and Physical Activity

As part of the postoperative follow-up monitoring, body weight was regularly recorded, resulting in an average increase of 6.2 ± 4.2 kg over the 16-week study with no difference between the treatment groups. Similarly, no significant changes were observed in the body condition score after surgery, with an average of 3.5 arbitrary units. By day 10 postsurgery, all sheep in the predicate and empty defect groups scored 0 on the posture and locomotion scale and no longer required any analgesia. All sheep from the chitosan group still exhibited lameness score of 3 and pain requiring additional analgesia. Lameness peaked on day 11 postop and with increased analgesia, started to improve after day 23 postop for all but one animal, which plateaued and had to be sacrificed at day 29 postop since she did not respond to the analgesic treatment at the pace stipulated in the project license. By day 33 postsurgery, all other animals in the chitosan group returned to a posture and locomotion score of 0 with no further requirement for analgesia.

An activity tracker positioned on the collar of all animals was used to assess body movements in the study. Recordings were analyzed considering three 48-h monitoring periods: immediately after surgery (d1–d2, early point), at 8 weeks (midpoint), and at 16 weeks (end of the study) were used. Data were differentiated based on day-time (6 am to 6 pm) and night-time (6 pm to 6 am), which recorded lower activity in line with the sleep/wake cycle. Day-time recordings showed significantly higher body movements for the empty group compared to the predicate and chitosan groups in the early phase of the study, while no differences were detected between groups at midpoint. At the end point, however, a significant difference was detected between the chitosan and empty groups (Figure 3A). For night-time recordings, no significant differences were observed between groups at early and study end point, while at midpoint, values recorded for the chitosan group were significantly higher in comparison to the empty group (Figure 3B).

### Macroscopic Observation of the Defects

At the end point (16 weeks), the left operated legs of all animals included in the study were assessed by macroscopic scoring in order to evaluate the surface of the defect (Figure 1).

**Figure 1:**
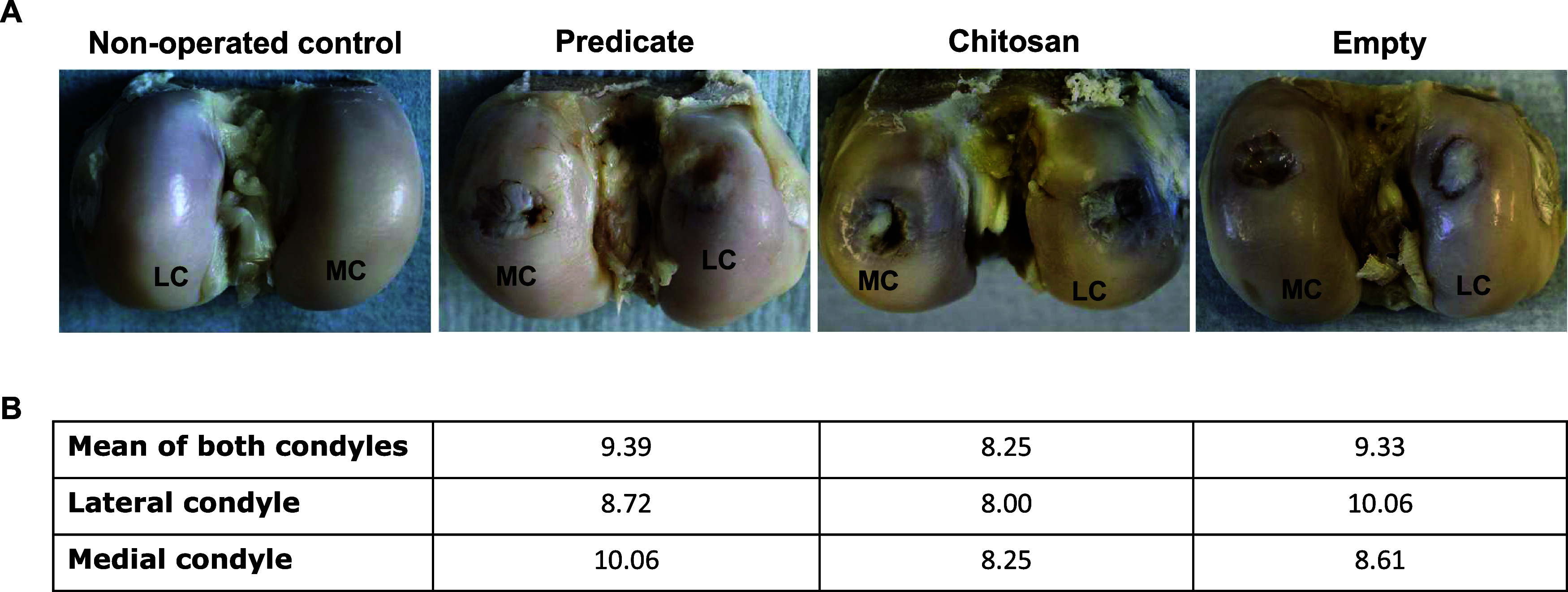
(A) Representative images of *ex vivo* bone condyles from all the groups of study (left knee) and nonoperated control group (right knee) at the study end point (16 weeks). MC = Medial condyle; LC = lateral condyle. (B) Macroscopic scoring of different parameters indicative of regeneration (expressed as arbitrary units based on criteria defined in Table 1).

A layer of new cartilage could be observed in all defects being in full (predicate) or partial (chitosan and empty) contact with the surrounding endogenous tissue. The presence of depressions and fissures was observed in the cartilage, indicating a certain degree of heterogeneity (Figure 1A). The macroscopic scoring of different parameters indicative of regeneration did not show significant differences between the three groups that scored 8.25 (chitosan), 9.39 (predicate), and 9.33 (empty) over a maximum score of 16 (Figure 1B).

### Evaluation of the Implant Site through Magnetic Resonance Imaging and Microcomputed Tomography

MRI and micro-CT scans were performed on fixed bone samples to evaluate the tissue formed in the defect after 16 weeks (Figure 2). MRI scans of samples from the predicate and chitosan groups showed the presence of a cartilage-like layer covering the defect (Figure 2A,B). In both groups, the layer appeared to cover the defect with no significant gaps and looked of a similar thickness to the endogenous layer of cartilage. In the empty group, the organization and structure of the superficial layer were variable across the samples; while one sample presented a good level of regeneration (even layer with thickness similar to the endogenous tissue), in 4 out of 6 samples, the cartilage layer was either absent or limited to a small marginal region, possibly originating from the inward growth of endogenous tissue.

**Figure 2:**
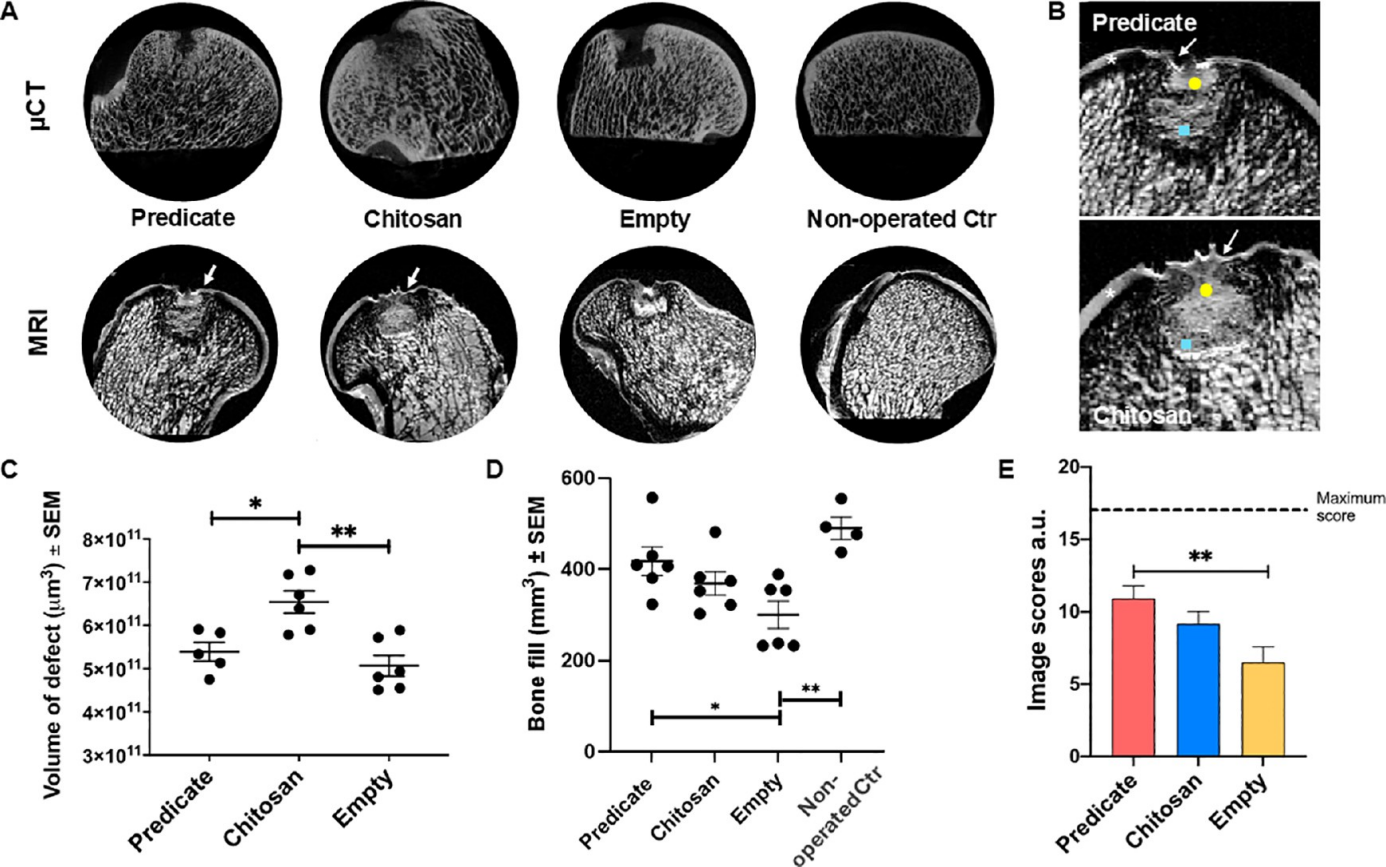
Magnetic resonance imaging and microcomputed tomography analysis of tissue regeneration. (A, B) Representative images of micro-CT and MRI scans of bone condyles of the three groups of study and nonoperated control at the study end point. White arrows in the predicate and chitosan group images point at the newly formed cartilage-like layer (white arrow), with a zoomed-in view in panel (B). White asterisk: old cartilage, yellow dot: remaining implant material, blue square: new bone formation. (C, D) Defect volume (C) and bone volume fill (D) at the study end point. (E) Degree of defect regeneration inferred from micro-CT and MRI results scored according to the system shown in Table 2, with maximum score assignable indicated by the dotted line in panel (E). a.u. = arbitrary units. **p < 0.01.

Micro-CT scans showed the presence of new bone-like tissue in the majority of samples across the three groups, although in variable amounts. Samples from the predicate group showed new bone occupying most of the defect area from the bottom region to the subchondral region. Conversely, samples from the chitosan and empty groups presented new bone-like tissue ingrowth mainly in the bottom region or in the subchondral bone (Figure 2A). The volume of each defect quantified across the three groups showed higher values for samples of the chitosan group in comparison to the predicate and empty samples (Figure 2C), although the degree of bone fill was comparable between the two types of implants (Figure 2D). Only the difference in bone fill between the empty defect and the unoperated control reached statistical significance confirming that the empty defect was unable to recover as expected. MRI and micro-CT images were scored according to the scoring system presented in Table 2 in order to evaluate the degree and structure of the new tissue filling the defect. Higher scores were recorded for the samples of the predicate and chitosan groups in comparison to the empty group, reaching statistical significance between the predicate and empty groups (Figure 2E).

### Hematoxylin & Eosin Staining

Sample sections from the three study groups were stained with hematoxylin & eosin to evaluate the tissue within the osteochondral defects. Nonoperated samples (condyles from right leg) were included as controls (Figure 3). For the three treatment groups, the layer of tissue formed on top of the defect appeared to be anchored to the endogenous tissue on either side, presenting a rough appearance in the chitosan and empty groups with some vertical fissures, while a smoother appearance was observed in the predicate group. A tidemark was observed in samples from the predicate group that resembled the one visible in the nonoperated control group (Figure 3A, inserts). The core region of the empty group’s defects was mainly filled by fibrous tissue together with adipose tissue, while areas of new bone-like tissue were limited to the edges of the defect of the subchondral region (Figure 3B). By contrast, the amount of adipose tissue in the predicate and chitosan groups was minimum, which displayed remnants of the respective scaffold material throughout the defects, surrounded by fibrous or bone-like tissue (Figure 3B). All around the defect edges of both predicate and chitosan groups, the new tissue displayed physical continuity with the endogenous bone. In the empty group, this was confirmed mainly in the osteochondral region, while in depth, the defect was mainly filled by adipose tissue (Figure 3C). The hematoxylin & eosin staining also highlighted local tissue infiltration of inflammatory cells in predicate and chitosan product groups (Figure 3D). These included visible foreign body multinucleated giant cells (MNGCs) observed in proximity of residual scaffold fragments and areas of lymphocyte-like cell infiltration detected only in the predicate group.

**Figure 3:**
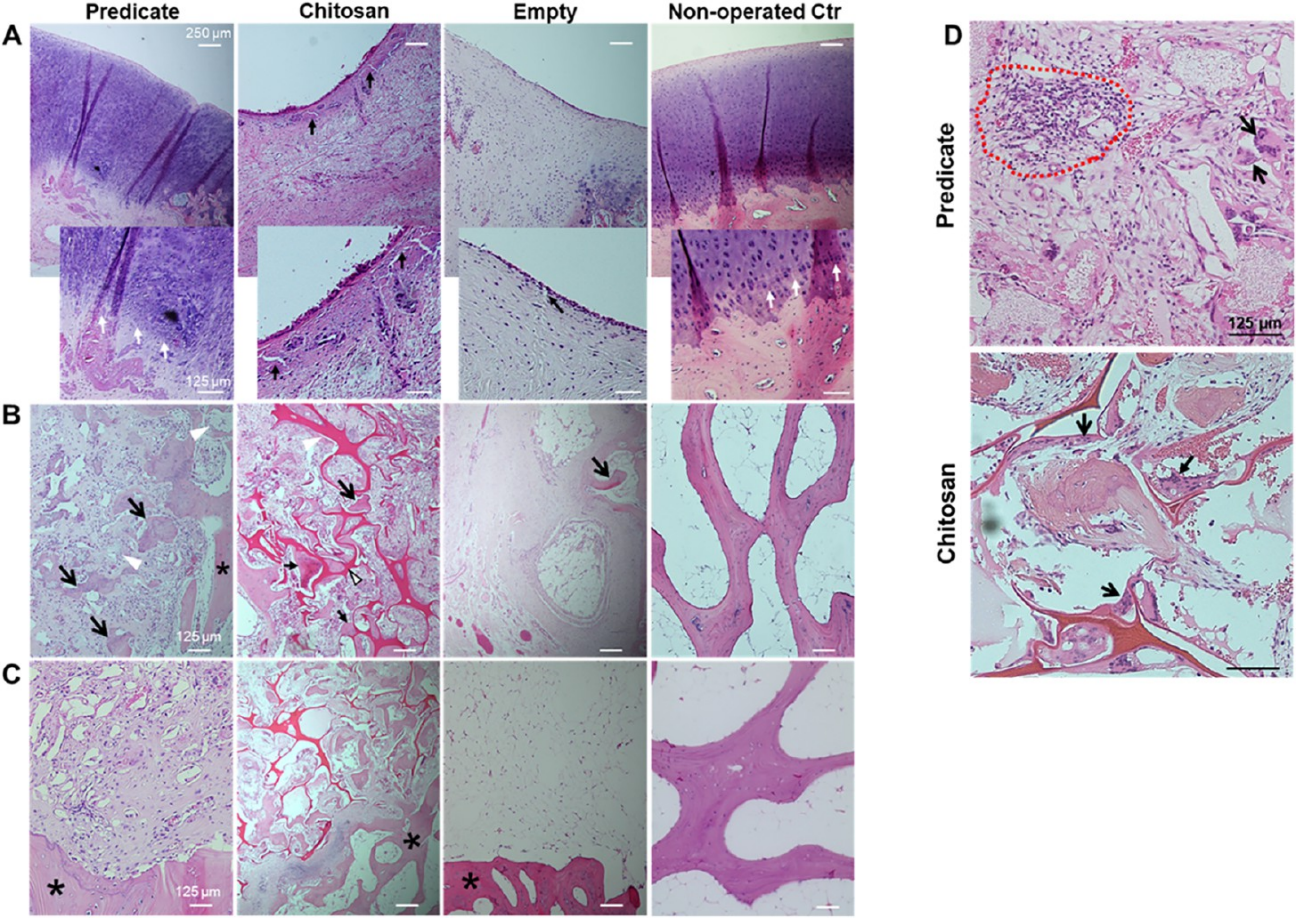
Eosin and hematoxylin staining of the tissue sections from three treatment groups and nonoperated control. (A) Representative images of the top layer of the defects and subchondral bone. Scale bar = 250 μm. Inserts at the bottom of each image show zoom-in views, with details of the tidemark structure (white arrows) observed in the predicate group and nonoperated control tissue. Black arrows show fissures in the cartilage layer. Scale bar = 125 μm. (B) Representative images of the central defect region for the three study groups and nonoperated control. Residual fragments of the scaffold visible in both the predicate and chitosan groups are indicated by white arrow heads. Black arrows indicate areas of new bone formation. Scale bar = 125 μm. (C) Representative images of the bottom part of the defects (predicate, chitosan, and empty groups) showing the interface between the endogenous bone (asterisk) and the new filling tissue. Scale bar = 125 μm. (D) Representative images of hematoxylin and eosin-stained sections showing local tissue infiltration of inflammatory cells including lymphocyte-like cells (red dotted line) seen in the predicate group and multinucleated giant cells (MNGCs) (black arrows) visible in both chitosan and predicate groups. Scale bar = 125 μm.

### Alcian Blue Staining and Cartilage Regeneration

Sections from L1 and L2 regions of all defects were stained for Alcian blue to visualize and analyze the newly regenerated cartilage layer (Figure 4). Samples from the predicate group presented an intense-blue staining (Figure 4A) with a mixed organization in the newly formed cartilage-like layer, while the staining in the chitosan group was lighter. Even more so for the empty group, which was pale and heterogeneous in both intensity and color spanning from light pink to light blue. Regarding the organization, this mainly resembled the fibrocartilage (Figure 4B).

**Figure 4:**
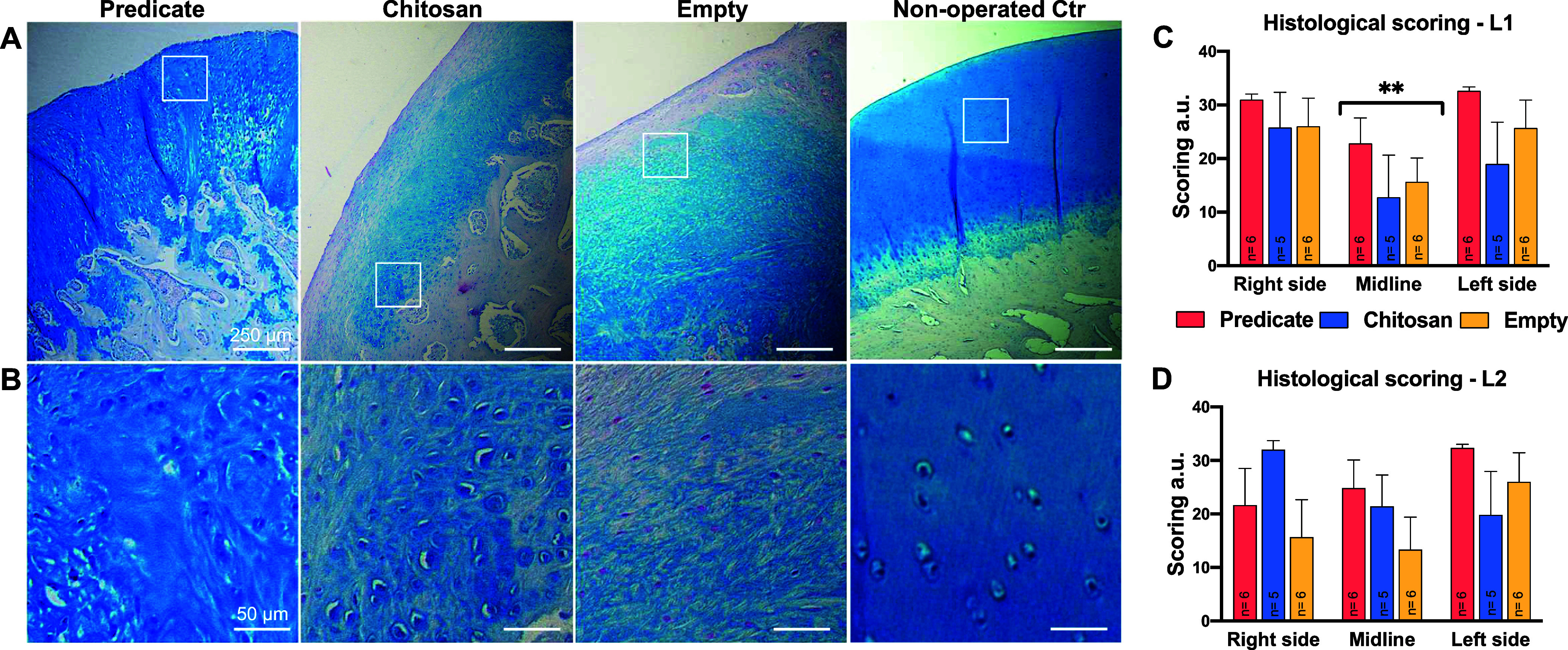
Alcian blue staining. Representative images of Alcian blue stained sections of the three groups of study and nonoperated control group. Images in panel (A) provide an overview of the top defect layer and the subchondral region. Inset areas are shown at higher magnification in panel (B). Scale bar = 250 μm. (B) Zoomed-in view of the boxed areas in panel (A) showing detailed cartilage layer nature and organization across the groups. Scale bar = 50 μm. (C, D) Histological scoring of new cartilage-like tissue performed on Alcian Blue stained sections from the L1 (C) and L2 (D) defect regions. “Right side”, “left side”, and “midline” indicate the relative areas of the defect selected for the scoring (see Figure S3). **p < 0.01: midline vs right and left sides.

Sections from the L1 and L2 regions of all defects were also scored according to criteria presented in Table 3 in order to evaluate the newly regenerated cartilage layer. The scoring was performed on three different areas of the cartilage including defect sides on the border with endogenous tissue and an area on the defect midline (Figure S2B).

Statistical analysis showed that both side areas of the L1 defect region (but not of the L2 region) obtained significantly higher scores than the midline area, while no differences were detected between the right and left side areas: the average values obtained were 27.71 au for the right area and 26.18 au for the left area (maximum score assignable: 36), and 17.35 for the midline region (maximum score assignable: 34). Within each of the 3 areas, a comparison between groups was also performed for each area (Figure 4C,D) and no significant differences were detected, although a trend of higher scores for the predicate groups could be identified for each area of the L1 region. High intergroup variability was observed for samples of the L2 defect region with neither significant differences nor a clear trend detectable.

### Masson’s Trichrome Staining and New Bone Formation

Masson’s trichrome staining was performed to evaluate the formation of new bone tissue within the defects (Figure 5). The predicate and chitosan defects contained numerous areas of newly formed bone, which were seen near the endogenous bone at the defect edge or in contact with the scaffold fragments (white arrow heads). In the predicate and chitosan groups, these new bone areas gave rise to visible interconnected trabeculae-like structures. Within these trabecular structures, the bone tissue presented the lamellar organization typical of mature bone with osteocytes embedded in the bone matrix. The defects of the empty group presented extended areas of fibrous tissue that in some cases could be juxtaposed to areas of new bone tissue organized in trabeculae. Interestingly, these areas of new bone tissue presented a nonhomogenous staining with greener staining on the border of the trabeculae and a paler color in the center (Figure 5).

**Figure 5:**
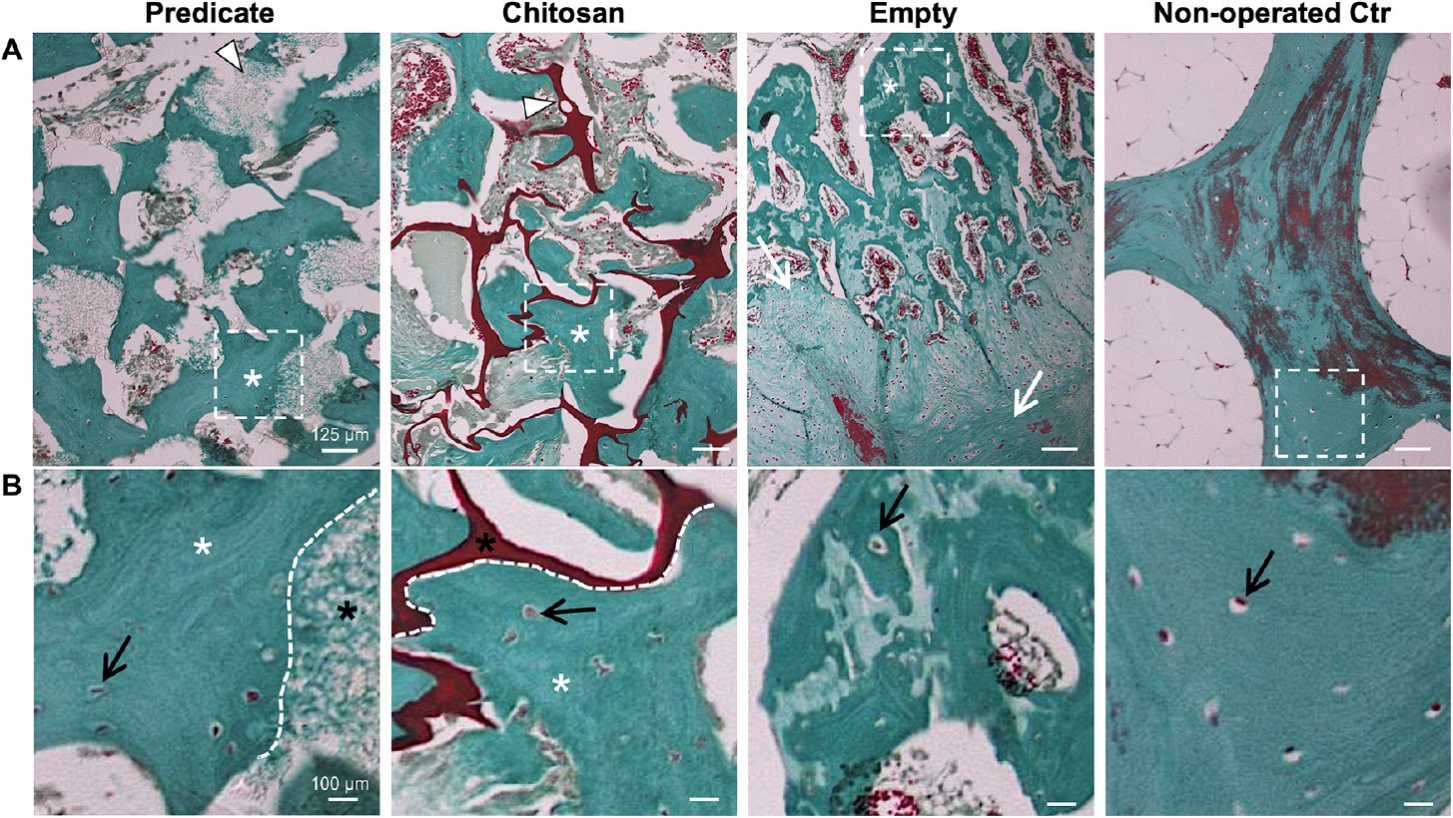
Masson’s trichrome staining of tissue sections from the three groups of study and nonoperated control. (A) White arrow heads point at residual fragments of predicate and chitosan scaffolds in the respective images; white asterisks indicate areas of new bone formation, while white arrows in the empty group image point at an area of fibrous tissue. The red-stained area in the nonoperated group corresponds to woven immature bone. Scale bar = 125 μm. (B) Zoomed-in view of the dotted inset areas in panel (A). Black arrows point at the osteocytes embedded in the bone-like tissue, and dotted lines show the border between the scaffold (black asterisk) and endogenous bone. Scale bar: 100 μm.

### CRP Measurement

Levels of C-reactive protein (CRP) were measured in serum samples of all animals at three time points (day 0, day 7, and study end point) to monitor signs of systemic inflammatory response during the period of study. No significant differences were detected by Elisa test semiquantitation between the groups of study at the three time points. The only significant variation observed was for the predicate group between days 0 and 7 (Figure 6).

**Figure 6:**
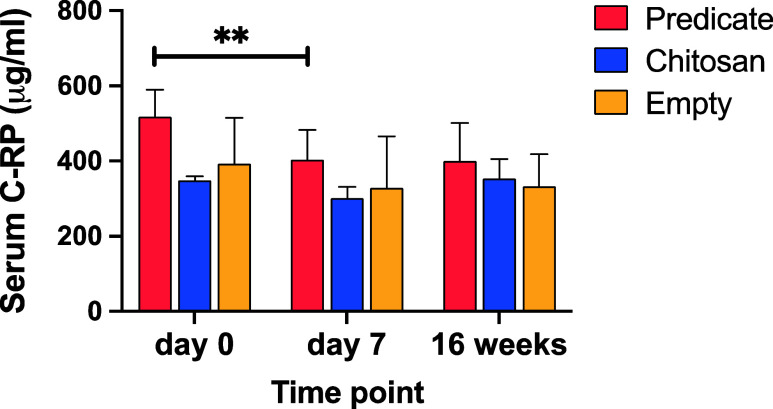
C-Reactive protein semiquantitation. C-Reactive protein measured through ELISA test in serum samples of the three groups of study at day 0, day 7 postimplantation, and study end point (16 weeks). **p < 0.01.

## Discussion

This study evaluated the biocompatibility and regenerative potentials of a chitosan-based biomimetic porous scaffold, shown in previous in vitro and ex vivo tests to support cell growth and differentiation and to be mechanically stable,^9,17^ in a large animal osteochondral model. Sheep was selected as a recognized relevant load-bearing model^18,19^ and, in order to minimize postoperative lameness by preserving one of the hindlegs, two defects were performed in both condyles of the left hind leg. A novel surgical approach was successfully carried out, which involved no luxation of the patella while accessing the condyles, in order to facilitate recovery and aid in avoiding the main postoperative complication of patella luxation. Indeed, this bicondylar defect approach produced a rapid recovery from surgery and did not lead to any issues with patella luxation occurring postoperatively in any of the sheep. The selected defect size (8 × 8 mm) exceeded the critical threshold as defined by previous studies, which reported a significantly reduced ability for endogenous healing for defects over 7 × 7 mm.^18,20,21^ In the present study, while defects from the empty group displayed a large proportion of adipose tissue fill, some of the samples also showed some spontaneous bone tissue regrowth in the defect but not on the cartilage surface. These observations suggest that the dimensions for a critical size osteochondral defect, which are also constrained by the configuration of the condyle, could be further optimized based on the strain and model considered.^22^ Another technical finding of this study arises from the interpretation of activity tracker data sets, and the possible interference of head movements, possibly related to pain or behavior, in the measurement of overall activity/mobility. When positioned on the collar, the trackers did not discriminate between movements performed when stationary or in motion, a challenge that could be resolved by positioning the trackers on the back of the animal rather than the neck region to exclude head movements. It may also be possible in the future to adjust the algorithm in order to extract a finer read-out of the animal’s locomotion movements.^23^

Here, a commercial predicate scaffold was implanted as a positive control of regeneration. Although such a scaffold is already approved for clinical use, it contains large animal-sourced collagen, which could raise some ethical and religious concerns in patients. Therefore, a collagen-free construct, such as the chitosan scaffold presented here, could constitute a valid alternative. Reports of chitosan-based material assessment in vivo are limited,^7^ although injectable chitosan products have been tested clinically in the knee in the context of osteoarthritis, with some signs of cartilage improvement.^24^ For the chitosan scaffold described here, the macroscopical observation and imaging of the implant sites at the end point did not show evidence of notable delamination of the chitosan product, in line with mechanical data gathered ex vivo in a previous gait simulation study.^17^ The osteochondral defect used in this study analyzed tissue regeneration at the level of both cartilage and the underlying osteochondral bone. Regarding the formation of new bone, all three experimental groups presented new bone-like tissue filling the deep region of the defect with trabecular-like structures and lamellar-like organization suggesting the formation of mature bone tissue. Even though the amounts of immature and mature bone-like tissues detected were not significantly different between the three groups, the majority of the empty defect group had large areas of adipose tissue occupying the osteochondral region. The presence and trabecular organization of the bone tissue is critical for correct bone functionality and effective support to the cartilage layer, and observations made here suggest that both chitosan and predicate osteochondral constructs had a positive effect on this aspect of tissue regeneration. Masson’s trichrome staining further confirmed the bone-like nature of the tissue as large, green-stained areas could be detected, indicating the presence of mineralized collagen-containing tissue. Conversely, red-stained areas were detected exclusively in the nonoperated control tissue, and this could be interpreted as part of physiological bone remodeling processes in normal healthy bones. The absence of such areas from the 3 intervention groups may be related to the timing of the analysis limited to 16 weeks, which represents an early time point for the onset of physiological bone remodeling.^25^ Regarding the formation of new cartilage-like tissue, this was generally poorer in comparison to the bone formation across the three groups. These observations reflect the difference in the spontaneous regenerative abilities of bone tissue, known to possess high spontaneous regenerative ability due to the presence of vascularization and innervation, and cartilage tissue, which displays poor regeneration capacity and is mainly avascular and noninnervate.^26^ Regarding the new cartilage formation, the best performance was observed for the predicate construct in comparison to the empty group and chitosan, as shown by the histological scoring and Alcian blue stained sections. The new cartilage-like tissue observed in the predicate group contained some hyaline-like cartilage mixed with fibrocartilage, while a lower amount of cartilage was observed for the chitosan and empty groups as fibrocartilage occupied most of the area. A different cartilage composition potentially affects the mechanical performance of the newly formed tissue. Indeed, the main function of the hyaline articular cartilage is to provide a smooth and lubricated surface to reduce the friction during transmission of mechanical loads within the articular joint. The composition of hyaline cartilage responds optimally to this goal as it is rich in water, collagen, and proteoglycans. Conversely, the fibrocartilage is a dense matrix rich in collagen-I and poor in proteoglycans and GAGs, making it mechanically disadvantageous in comparison to the hyaline one.^26^ For future investigations, the addition of a targeted immunohistochemical analysis surveying the expression of markers such as collagen type I and II should be included to provide further characterization of the presence and ratio of these two types of cartilage within the defects analyzed.

An important aspect of devices used for regenerative approaches is the possible stimulation of an inflammatory response at local or systemic levels. While a weak inflammatory response is generally associated with positive outcomes,^20^ a stronger response can potentially lead to the failure of the approach by altering the properties of the scaffolds or preventing its supporting interaction with the local tissue by encapsulating it in a fibrous capsule.^27^ In this study, during the first 4 weeks after implantation, animals that received the chitosan scaffold appeared to experience some discomfort alleviated with additional pain-relief treatment. It is worth mentioning that such a reaction reverted in the long term, and at the study end point, animals were fully recovered, as confirmed by the posture and locomotion scoring and the absence of C-reactive protein in serum samples. Interestingly, the local tissue reaction was considered weak as no lymphoid-like cell infiltration was observed, in contrast to what was observed in the predicate group, but only the presence of multinucleated cells resembling the Foreign Body Giant Cells. These cells possessing intrinsic phagocytic ability constitute a known physiological mechanism to remove foreign materials.^28,29^ The reason for the initial reaction, which might have been more acute due to the presence of 2 implants in the same joint, is not clear and could be linked to the composition of the chitosan powder used for manufacturing, such as the deacetylation degree or trace contamination with endotoxin.^30^ It could alternatively result from the release of degradation products during the breakdown of the material, observed in vitro to lead to an early and transient increase in glucosamine, which could cause a local peak possibly related to the temporary discomfort subsequently resolved in the chitosan group.^31^ Further studies are under way to identify the basis for this transient reaction and optimize the construct parameters for future applications, for instance, through the addition of a soaking step before implantation to offset the release of degradation products postoperation.

## Conclusions

This study confirmed that the implantation of a biomimetic construct into an osteochondral defect in sheep exerts a positive effect on regeneration. The novel surgical method developed here, allowing us to drill two defects into the same condyle without luxating the patella, appeared safe and well-tolerated by the animals, representing an advantage in terms of animal welfare and optimization of the number of animals used.

The trend observed of better regeneration for the predicate product confirms its role as positive control in the promotion of bone and cartilage regeneration, while the chitosan construct supported subchondral bone regeneration at levels similar to the predicate and was well-tolerated in the long term, despite a transient phase of apparent discomfort. Further studies are under way to clarify the causes of this initial reaction, in order to collect information on local tissue response and improve the scaffold’s biological properties. Regarding the more challenging regeneration of the cartilage layer, the delay observed in the degree of maturation in comparison to the predicate could be due to the short duration of this pilot study. These observations now warrant a follow-up trial analyzing the scaffold’s performance and integration at a longer time point in future studies, alongside a comprehensive analysis of the status of inflammatory cells and cytokines over the regeneration time-course. In addition, some further steps could be considered to improve the cartilage regeneration either through a preimplantation cellularization phase of the scaffold to initiate chondrogenesis ex vivo prior to the intervention, which may present some logistical challenges but also benefits in terms of clinical translation,^32^ or though the possible incorporation of prochondrogenic biologics such as TGFβ1 or kartogenin in the construct itself to locally promote cartilage formation.^7,33^

## Supplementary Material



## Data Availability

Data is available from the authors on reasonable request.

## Notes

Additional experimental details, including surgical, imaging, and monitoring setup (PDF)

## References

[1] Nukavarapu S. P., Dorcemus D. L. (2013). Osteochondral tissue
engineering: current strategies
and challenges. Biotechnol Adv..

[2] Fu L., Yang Z., Gao C., Li H., Yuan Z., Wang F. (2020). Advances and prospects
in biomimetic multilayered scaffolds
for articular cartilage regeneration. Regen.
Biomater..

[3] Zhang B., Huang J., Narayan R. J. (2020). Gradient scaffolds for osteochondral
tissue engineering and regeneration. J. Mater.
Chem. B.

[4] Nooeaid P., Salih V., Beier J. P., Boccaccini A. R. (2012). Osteochondral
tissue engineering: scaffolds, stem cells and applications. J. Cell Mol. Med..

[5] Longley R., Ferreira A. M., Gentile P. (2018). Recent Approaches
to the Manufacturing
of Biomimetic Multi-Phasic Scaffolds for Osteochondral Regeneration. Int. J. Molecular Sci..

[6] Oryan A., Sahvieh S. (2017). Effectiveness of chitosan scaffold in skin, bone and
cartilage healing. Int. J. Biol. Macromol..

[7] Rawojć K., Tadeusiewicz R., Zych-Stodolak E. (2025). Advancements in Chitosan-Based Scaffolds
for Chondrogenic Differentiation and Knee Cartilage Regeneration:
Current Trends and Future Perspectives. Bioengineering.

[8] Kheirabadi B. S., Acheson E. M., Deguzman R., Sondeen J. L., Ryan K. L., Delgado A. (2005). Hemostatic
efficacy of two advanced dressings in an
aortic hemorrhage model in Swine. J. Trauma..

[9] Pitrolino K. A., Felfel R. M., Macri Pellizzeri L., McLaren J., Popov A. A., Sottile V., Scotchford C. A., Scammell B. E., Roberts G. A. F., Grant D. M. (2022). Development and in vitro assessment of a bi-layered
chitosan-nano-hydroxyapatite osteochondral scaffold. Carbohydr. Polym..

[10] Kandel R. A., Grynpas M., Pilliar R., Lee J., Wang J., Waldman S. (2006). Repair of osteochondral
defects with biphasic cartilage-calcium
polyphosphate constructs in a sheep model. Biomaterials.

[11] Carson J. S., Bostrom M. P. (2007). Synthetic bone scaffolds and fracture repair. Injury.

[12] Millán D., Jimenez R. A., Nieto L. E., Poveda I. Y., Torres M. A., Silva A. S. (2021). Adjustable conduits
for guided peripheral nerve
regeneration prepared from bi-zonal unidirectional and multidirectional
laminar scaffold of type I collagen. Mater.
Sci. Eng. C Mater. Biol. Appl..

[13] Goebel L., Orth P., Muller A., Zurakowski D., Bucker A., Cucchiarini M. (2012). Experimental scoring
systems for macroscopic articular cartilage repair correlate with
the MOCART score assessed by a high-field MRI at 9.4 T--comparative
evaluation of five macroscopic scoring systems in a large animal cartilage
defect model. Osteoarthritis Cartilage.

[14] van
den Borne M. P., Raijmakers N. J., Vanlauwe J., Victor J., de Jong S. N., Bellemans J., Saris D. (2007). International Cartilage
Repair Society (ICRS) and Oswestry macroscopic cartilage evaluation
scores validated for use in Autologous Chondrocyte Implantation (ACI)
and microfracture. Osteoarthritis Cartilage.

[15] Hossain K. M. Z., Patel U., Kennedy A. R., Macri-Pellizzeri L., Sottile V., Grant D. M. (2018). Porous calcium phosphate
glass microspheres for orthobiologic applications. Acta Biomater..

[16] MLaren J. S., Macri-Pellizzeri L., Hossain K. M. Z., Patel U., Grant D. M., Scammell B. E. (2019). Porous Phosphate-Based
Glass Microspheres Show
Biocompatibility, Tissue Infiltration, and Osteogenic Onset in an
Ovine Bone Defect Model. ACS Appl. Mater. Interfaces.

[17] Cowie R. M., Macri-Pellizzeri L., McLaren J., Sanderson W. J., Felfel R. M., Scotchford C. A., Scammell B. E., Grant D. M., Sottile V., Jennings L. M. (2024). Functional
performance of a bi-layered
chitosan-nano-hydroxyapatite osteochondral scaffold: a pre-clinical
in vitro tribological study. R Soc. Open Sci..

[18] Meng X., Ziadlou R., Grad S., Alini M., Wen C., Lai Y., Qin L., Zhao Y., Wang X. (2020). Animal Models of Osteochondral
Defect for Testing Biomaterials. Biochem Res.
Int..

[19] Oláh T., Cai X., Michaelis J. C., Madry H. (2021). Comparative anatomy and morphology
of the knee in translational models for articular cartilage disorders.
Part I: Large animals. Ann. Anat..

[20] Cook J. L., Hung C. T., Kuroki K., Stoker A. M., Cook C. R., Pfeiffer F. M. (2014). Animal
models of cartilage repair. Bone Joint Res..

[21] Lietman S. A., Miyamoto S., Brown P. R., Inoue N., Reddi A. H. (2002). The temporal
sequence of spontaneous repair of osteochondral defects in the knees
of rabbits is dependent on the geometry of the defect. J. Bone Joint Surg British.

[22] Lydon H., Getgood A., Henson F. M. D. (2019). Healing
of Osteochondral Defects
via Endochondral Ossification in an Ovine Model. Cartilage.

[23] Casilari E., Barbosa-Galeano J., González-Cañete F. J. (2024). UMAHand:
A dataset of inertial signals of typical hand activities. Data Brief..

[24] De
Marziani L., Boffa A., Andriolo L., Di Martino A., Filardo G., Zaffagnini S. (2024). Chitosan-based scaffold augmentation
to microfractures: Stable results at mid-term follow-up in patients
with patellar cartilage lesions. J. Exp. Orthop..

[25] Dimitriou R., Jones E., McGonagle D., Giannoudis P. V. (2011). Bone regeneration:
current concepts and future directions. BMC
Med..

[26] Sophia
Fox A. J., Bedi A., Rodeo S. A. (2009). The basic science
of articular cartilage: structure, composition, and function. Sports Health.

[27] Vasconcelos D. P., Aguas A. P., Barbosa M. A., Pelegrin P., Barbosa J. N. (2019). The inflammasome
in host response to biomaterials: Bridging inflammation and tissue
regeneration. Acta Biomater..

[28] Barbeck M., Booms P., Unger R., Hoffmann V., Sader R., Kirkpatrick C. J., Ghanaati S. (2017). Multinucleated giant cells in the
implant bed of bone substitutes are foreign body giant cells-New insights
into the material-mediated healing process. J. Biomed Mater. Res. A.

[29] Milde R., Ritter J., Tennent G. A., Loesch A., Martinez F. O., Gordon S. (2015). Multinucleated
Giant Cells Are Specialized for Complement-Mediated
Phagocytosis and Large Target Destruction. Cell
Rep..

[30] Fong D., Hoemann C. D. (2018). Chitosan immunomodulatory properties:
perspectives
on the impact of structural properties and dosage. Future Sci. OA.

[31] Pitrolino K., Felfel R., Roberts G., Scotchford C., Grant D., Sottile V. (2024). In vitro degradation of a chitosan-based
osteochondral construct points to a transient effect on cellular viability. Biomed Mater..

[32] Chen C. P., Weng P. W., Lee K. T., Chiang L. Y., Liao W. J., Shaw L. (2025). Biphasic Scaffold Loaded With Autologous Cartilage Yields Better
Clinical Outcome and Magnetic Resonance Imaging Filling Compared With
Marrow Stimulation for Focal Osteochondral Lesions in the Knee. Arthroscopy.

[33] Fang C. H., Lin Y. W., Sun C. K., Sun J. S. (2023). Small-Molecule
Loaded
Biomimetic Biphasic Scaffold for Osteochondral Regeneration: An In
Vitro and In Vivo Study. Bioengineering.

