# Supplementary material for: Implantation and Analysis of a Biphasic Bioinspired Osteochondral Chitosan Construct in a Large Animal Model
Source: ACS Biomater Sci Eng. 2026 Mar 13;12(4):2315–25. doi: 10.1021/acsbiomaterials.5c01821 (PMC13080774; doi:10.1021/acsbiomaterials.5c01821)
Supplement: Supplementary file 1 [file ab5c01821_si_001.pdf]

Implantation and analysis of a biphasic bioinspired osteochondral chitosan construct in a large animal model.

Laura Macri Pellizzeri[1#], Jane S. McLaren[1#], Reda Felfel[2,3,4], Katherine Pitrolino[1,5], Robert Kerslake[1], Colin Scotchford[2], David M. Grant[2], Brigitte S. Scammel[1], Virginie Sottile[6,7*].

[1] School of Medicine, University of Nottingham, Nottingham, NG7 2UH, UK
[2] Advanced Materials Research Group, Faculty of Engineering, University of Nottingham, Nottingham, NG7 2RD, UK
[3] Department of Mechanical and Aerospace Engineering, University of Strathclyde, Glasgow, G1 1XJ, UK
[4] Department of Physics, Faculty of Science, Mansoura University, Mansoura, 35516, Egypt
[5] Research and Innovation Office, Loughborough, LE11 3TU, UK
[6] Department of Molecular Medicine, University of Pavia, Pavia, 27100, Italy
[7] UOC Bioscaffolds and transplantation, Fondazione IRCCS Policlinico San Matteo, Pavia, 27100, Italy

[#] These authors contributed equally to this work.

Suppl. Fig. S1

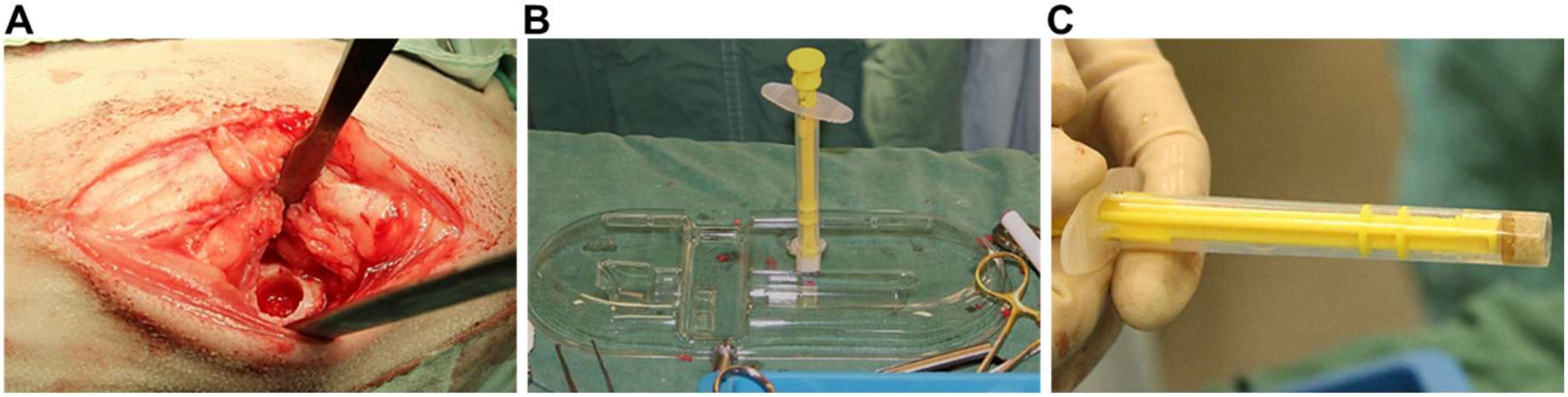

Suppl. Fig. S1. Surgical implant model. **(A)** 8x8mm cylindrical defects were created into both condyles of left hind legs. **(B)** Preparation of the Predicate scaffold into the delivery device. **(C)** Delivery device loaded with chitosan scaffold.

Suppl. Fig. S2

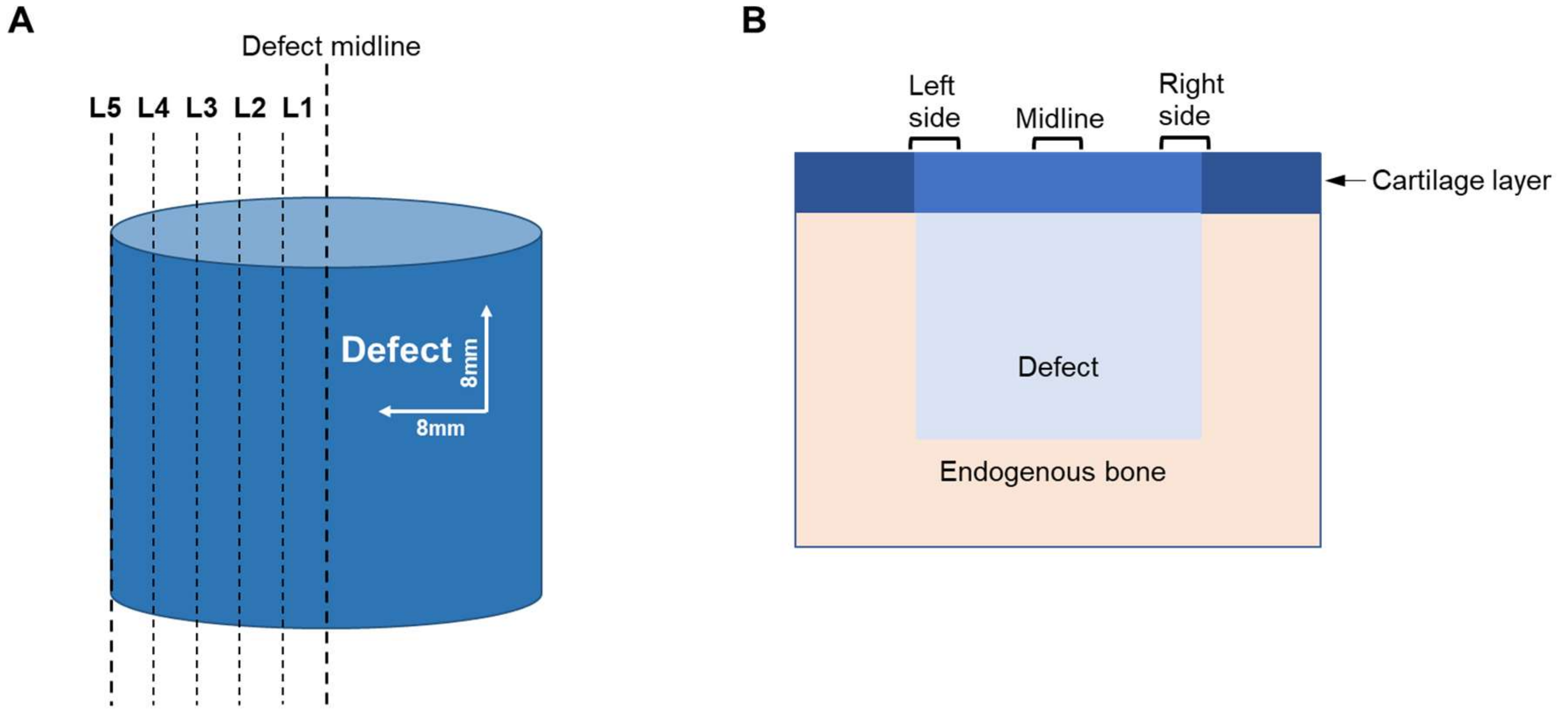

Suppl. Fig. S2: Schematic representation of the defect partition for histological processing and analysis. **(A)** Defects halved across the mid lane in the longitudinal plane were sectioned to obtain 5 regions (L1-5), from which 5 µm slices were produced for histological analysis. **(B)** Position of the three areas selected for the histological scoring of the new cartilage-like layer. Left and right side areas were selected to include the border with the endogenous tissue in order to evaluate the physical continuity with endogenous cartilage.

Suppl. Fig. S3

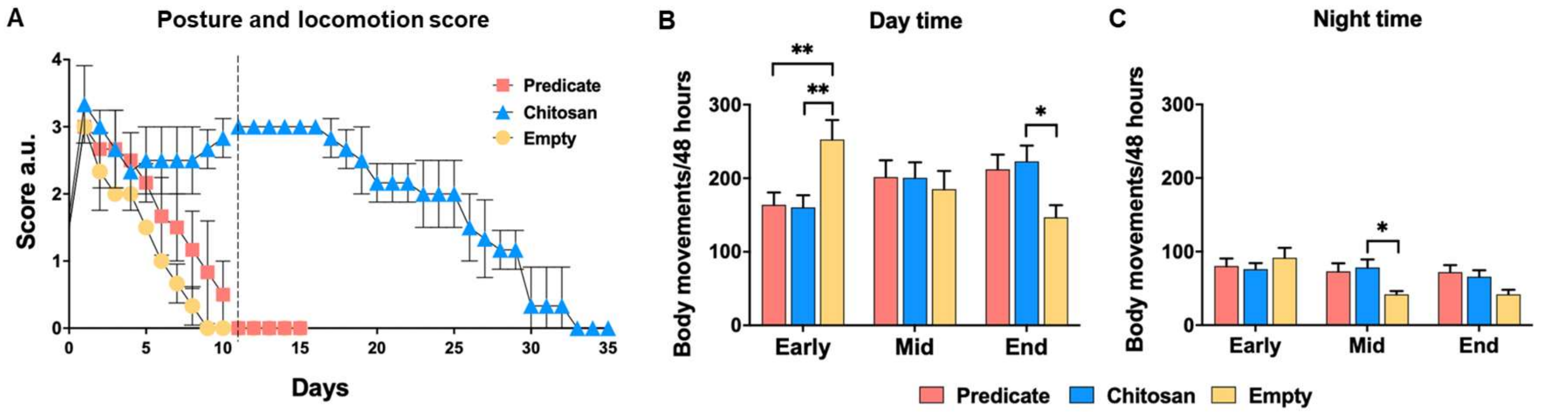

Suppl. Fig. S3. Posture and locomotion score **(A)** and animal movements recorded during 48h at early (d1-d2 post-surgery), mid (8 weeks) and end point of the study (16 weeks), during day (6am-6pm) **(B)** and night time (6pm-6am) **(C)**. ***p*< 0.01*;* **p*< 0.05*.*
